# The biological alterations of synapse/synapse formation in sepsis-associated encephalopathy

**DOI:** 10.3389/fnsyn.2022.1054605

**Published:** 2022-12-02

**Authors:** Chuan Tang, Ye Jin, Huan Wang

**Affiliations:** College of Life and Health, Dalian University, Dalian, China

**Keywords:** sepsis-associated encephalopathy, synapse formation, synaptic dysfunction, synaptic proteins, synaptic adhesion molecules, neuroinflammation

## Abstract

Sepsis-associated encephalopathy (SAE) is a common complication caused by sepsis, and is responsible for increased mortality and poor outcomes in septic patients. Neurological dysfunction is one of the main manifestations of SAE patients. Patients may still have long-term cognitive impairment after hospital discharge, and the underlying mechanism is still unclear. Here, we first outline the pathophysiological changes of SAE, including neuroinflammation, glial activation, and blood-brain barrier (BBB) breakdown. Synapse dysfunction is one of the main contributors leading to neurological impairment. Therefore, we summarized SAE-induced synaptic dysfunction, such as synaptic plasticity inhibition, neurotransmitter imbalance, and synapses loss. Finally, we discuss the alterations in the synapse, synapse formation, and mediators associated with synapse formation during SAE. In this review, we focus on the changes in synapse/synapse formation caused by SAE, which can further understand the synaptic dysfunction associated with neurological impairment in SAE and provide important insights for exploring appropriate therapeutic targets of SAE.

## Introduction

Sepsis is a severe pathological syndrome that was the most common cause of inpatient death worldwide in 2017, with 48.9 million cases and 11 million deaths (Rudd et al., 2020). Sepsis is caused by a dysregulated host response to infection and remains the leading cause of intensive care unit (ICU) morbidity and mortality worldwide (Seymour et al., 2016; Singer M. et al., 2016). Sepsis is represented by an imbalance in the pro-inflammatory and anti-inflammatory factors, leading to injury, multiple organ dysfunction (MOD), and failure. The central nervous system (CNS) is one of the first organs affected, and its clinical manifestation is sepsis-associated encephalopathy (SAE) (Dellinger et al., 2013; Schulte et al., 2013; Mazeraud et al., 2016; Shankar-Hari et al., 2016). SAE is a common complication in patients with sepsis, which seriously affects the prognosis and quality of life (Crippa et al., 2018). SAE patients usually present with severe systemic infection (without direct brain infection) with features of systemic inflammatory response syndrome (SIRS) (Gofton and Young, 2012). SAE is clinically characterized by slowing speed of information processing, impaired attention, memory dysfunction, delirium, or coma (Gofton and Young, 2012; Fernando et al., 2018; Moraes et al., 2021). The main feature of SAE is the change of electroencephalogram (EEG), and nonconvulsive status epilepticus appeared in 20% of cases (Hosokawa et al., 2014). In addition, SAE is also associated with psychological disorders, including anxiety, depression, and post-traumatic stress disorder (PTSD) (Hosokawa et al., 2014; Righy et al., 2019).

Despite all the efforts to improve SAE treatment in the last decades, there is still a lack of specific therapy for SAE in the clinic, and the mortality rate remains high. Although many pharmacological treatment strategies have been studied, researchers have found that many drugs commonly used in the ICU (including anticholinergic and histamine drugs) can trigger neurotoxic side effects, such as treating ICU patients with rivastigmine (a cholinesterase inhibitor) can lead to higher mortality rates (Campbell et al., 2009; Van Eijk et al., 2010). Therefore, there is no treatment or prophylaxis for SAE to date. The fundamental reason for the lack of effective treatment strategies is that the mechanisms of SAE have not been clarified. Numerous shreds of evidence suggest that the mechanisms involved in SAE pathology are diverse, including neuroinflammation, glia activation, changes in the blood–brain barrier (BBB) permeability, oxidative stress due to mitochondrial dysfunction, neurotransmission dysfunction, and synaptic plasticity change (Stubbs et al., 2013; Czempik et al., 2020; Li Y. et al., 2020; Moraes et al., 2021). A recent work is to analyze the proteomics of cerebrospinal fluid (CSF) samples from neonates with clinical sepsis. The results show that the abundance of proteins is decreased and associated with axonal and synaptic network development. These changes in protein in CSF may be closely related to sepsis-induced neuroinflammation and affect the formation of neural circuits (Jiang et al., 2022). Studies of human synaptic proteome and genome sequencing have shown that disruption of synaptic proteins can produce mutations and cause about 130 brain diseases (Jamjoom et al., 2021). Therefore, the abnormal level of synaptic proteins may be one of the reasons for many brain diseases. Although most studies on SAE have focused on neuronal injury, it is hypothesized that synapses and their related molecules may also be involved in the pathology of SAE, considering their important role in various brain diseases. Moreover, it has been shown that if synapse formation and maintenance are abnormal, it will lead to a series of serious neurological diseases such as autism, schizophrenia, and Alzheimer's disease (Blanco-Suarez et al., 2017). Then, the sequelae, such as neurosis and depression caused by SAE, may be related to changes in synapse formation. Since alterations in synapse and synapse formation may be the major basis for trying to explain brain dysfunction caused by neural transmission abnormality, in this review, we mainly discuss the alterations of synapses/synapse formation during SAE.

## Pathogenesis/pathophysiology of SAE

The pathophysiology of SAE remains incompletely understood. SAE has a variety of pathological mechanisms, including neuroinflammation, glia activation and BBB vascular changes leading to direct neural damage (Mazeraud et al., 2016, 2020; Tauber et al., 2017). It seems that neuroinflammation is the starting point of brain functional disturbance since systemic inflammation leads to profound alterations in cerebral homeostasis in SAE. Neuroinflammation plays a crucial role in neural apoptosis and cognitive impairment during SAE (Calsavara et al., 2018; Tian et al., 2019). Therefore, neuroinflammation may be a primary indicator of SAE. Under physiological conditions, the BBB is essential for the maintenance of homeostasis enabling normal neuronal function (Abbott et al., 2010; Nwafor et al., 2019). Disruption of BBB integrity has been consistently observed in SAE animal experiments (Flierl et al., 2009; Singer B. H. et al., 2016; Varatharaj and Galea, 2017). It is reported that the downregulation of tight junction proteins (e.g., occludin, ZO-1, and claudin) can be observed in brain tissue samples from septic patients who died, which may be responsible for BBB damage (Erikson et al., 2020). Due to the BBB breakdown, magnetic resonance imaging (MRI) showed cytotoxic edema, vasogenic edema, and high intensity of white matter in patients with SAE survivals (Stubbs et al., 2013; Manabe and Heneka, 2022). Impaired BBB function contributes greatly to the pathological progress of SAE, as the CNS becomes highly vulnerable to neurotoxic factors such as free radicals, inflammatory mediators, and intravascular proteins and leukocytes (Handa et al., 2008; Gao and Hernandes, 2021). Consequently, BBB deficiency causes the leakage of intravascular proteins and plasma into the extravascular space and leads to brain edema and reduced microvascular perfusion, contributing to exacerbating neuronal loss in SAE (Van Der Poll et al., 2017). In sepsis, a large number of uncontrolled inflammatory factors are produced, often referred to as a “cytokine storm” or “cytokine cascade” (although there is no clear definition), thus exhibiting an acute inflammatory response and even organ dysfunction. During sepsis, these pro-inflammatory cytokines, such as tumor necrosis factor- alpha (TNF-α) and interleukins-1β (IL-1β), are able to activate endothelial cells. Activation of endothelial cells leads to the production of reactive oxygen species (ROS) and nitric oxide (NO), thus increasing the BBB permeability (Mayhan, 1998; Handa et al., 2008; Sharshar et al., 2010). In sepsis, brain damage is further exacerbated by the interplay between abnormal endothelial cells and BBB breakdown, microglia activation, and neutrophil infiltration (Wang et al., 2015). In addition, endothelial injury can trigger disturbances in brain perfusion, leading to abnormal cellular metabolism and oxidative stress, which makes cerebral ischemia a problem that cannot be ignored in the course of the SAE (Taccone et al., 2013).

Once BBB permeability is compromised, microglia can be damaged and become active (Da Fonseca et al., 2014). Increased neuronal apoptosis associated with microglial activation was also specifically demonstrated in the brain of patients who died of sepsis (Sharshar et al., 2003). Studies have confirmed that sepsis-induced excessive activation of microglia can further worsen the BBB and participate in the progression of brain dysfunction (Michels et al., 2014, 2015). Conversely, inhibition of microglia can reduce brain damage and inflammation in sepsis while improving long-term cognitive function (Michels et al., 2015). Additionally, astrocytes control the BBB permeability and secrete inflammatory mediators, further facilitating the development of neuroinflammation, regulating the concentration of neurotransmitters such as glutamate, γ Aminobutyric acid (GABA), and glycine in the synaptic cleft (Seifert et al., 2006; Retamal et al., 2007; Howarth, 2014). In SAE animal experiments, astrogliosis can be observed (Moraes et al., 2015; Singer M. et al., 2016). The consequence of astrocyte proliferation or astrogliosis is the inability to regulate neurotransmitters, which will lead to glutamate toxicity (Heneka et al., 2015). Besides, astrocytes exhibit loss of end feet and structural remodeling after lipopolysaccharide (LPS) administration, which is responsible for the BBB disruption (Cardoso et al., 2015).

In conclusion, dysfunction of vascular complexes including endothelial cells, astrocytes, and BBB and activation of microglia can lead to neuroinflammation and add to neurotoxicity, hypoxia and induce metabolic stress (Mazeraud et al., 2020). During SAE, activation of microglia and astrocytes leads to neuroinflammation, increased excitotoxicity and metabolic imbalance, resulting in neuronal cell death (Mazeraud et al., 2020). Moreover, neuroinflammation affects cellular metabolism and may lead to oxidative stress related to mitochondrial dysfunction, thus changing neuronal function and viability (Semmler et al., 2007; Taccone et al., 2010). Mitochondrial dysfunction associated with the mitochondrial respiratory chain activity has been found in rat brains of the SAE (Comim et al., 2008). And, the mitochondrial dysfunction can lead to neuronal death in septic animal (Li Y. et al., 2020).

**Figure 1:**
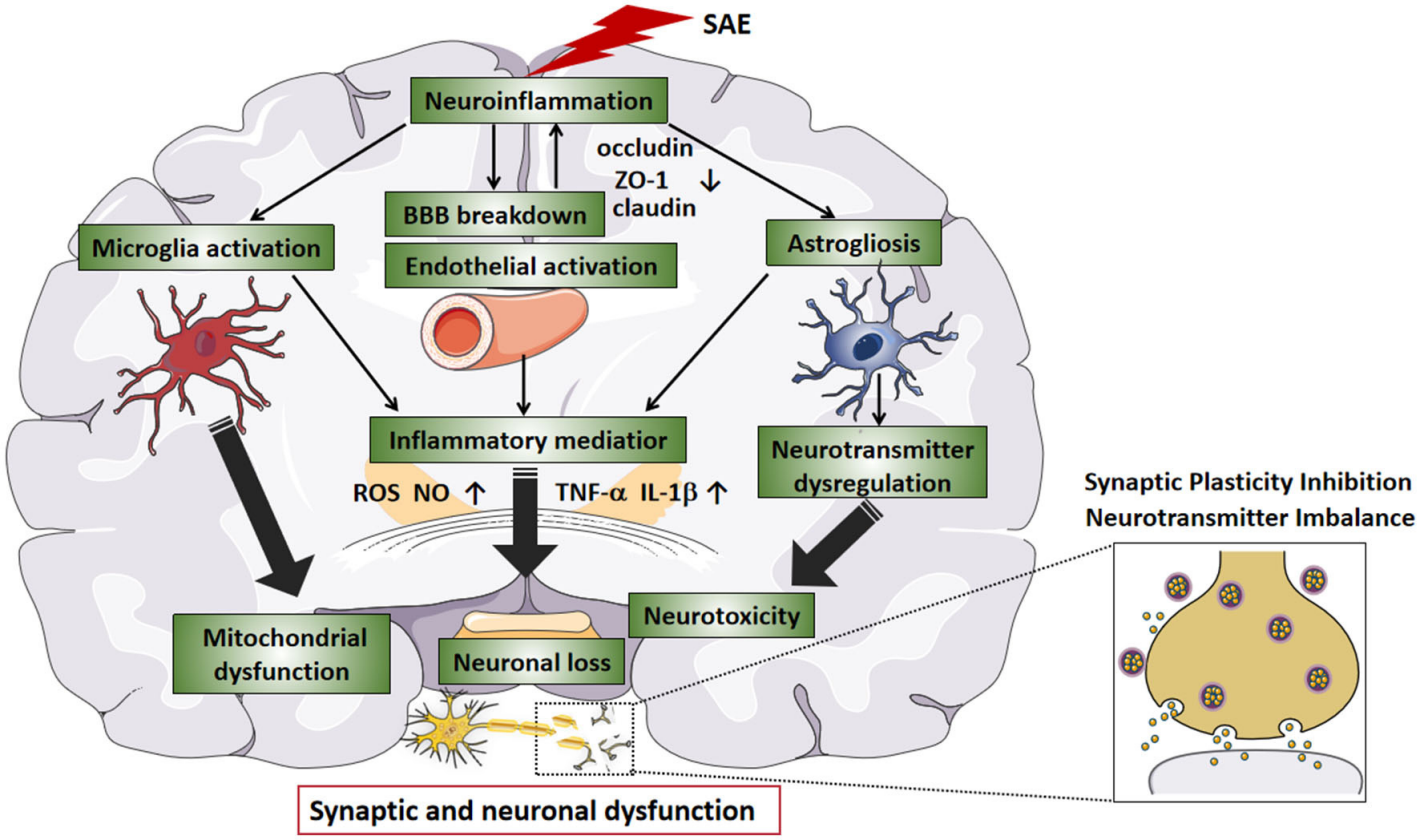
The pathophysiological mechanisms in SAE. Neuroinflammation is considered to be the main indicator of SAE. During SAE, downregulation of tight junction proteins (e.g., occludin, ZO-1, and claudin) marks BBB disruption. As the BBB breakdown, intravascular material leaks outside the blood vessels, causing cerebral edema and reduced microvascular perfusion, which leads to neuronal loss. In addition, the release of large amounts of inflammatory factors (e.g., TNF-a and IL-1b) leads to an acute inflammatory response and activates endothelial cells, producing ROS and NO, further increasing BBB permeability. Endothelial injury causes abnormal cellular metabolism, oxidative stress, and disturbance of brain perfusion, leading to ischemia. After SAE, hyperactivation of microglia can further exacerbate the inflammatory response, and BBB leakage, and participate in the progression of brain dysfunction. Activated astrocytes produce pro-inflammatory cytokines that exacerbate neuroinflammatory responses, produce excitotoxicity, and lead to neuronal death. SAE-induced dysfunction of the vascular complex (including endothelial cells, astrocytes, and BBB) and activation of microglia can exacerbate neuroinflammation, increase neurotoxicity and hypoxia and lead to mitochondrial dysfunction, thereby exacerbating neurological damage. In SAE, the interaction between neuroinflammation, glial cell activation, and BBB injury ultimately affects synaptic and neuronal dysfunction. SAE, sepsis-associated encephalopathy; BBB, blood-brain barrier; TNF-a, tumor necrosis factor-alpha; IL-1b, interleukin-1b; ROS, reactive oxygen species; NO, nitric oxide.

As mentioned earlier, excessive release of cytokines can reduce the concentration of protein C and activated protein C and, together with endothelial vascular damage, tilt the balance of the hemostatic system in the direction of coagulation and microthrombosis, leading to ischemia during sepsis (Cepinskas and Wilson, 2008). Ischemic lesions can often be observed in SAE patients or animals, which may contribute to neuronal apoptosis or neuronal loss. Taken together, neuroinflammation, glial cell activation, BBB damage and oxidative stress play an important role in the pathogenesis of SAE. However, researchers cannot determine which mechanism is the most important, so they speculate that their roles may be intertwined. The etiology of SAE is likely multi-factorial, and a number of pathomechanisms are involved in parallel, influence each other, and contribute to a varying degree to the development of SAE. The final result of these processes may be altered neurotransmission and neuronal dysfunction, leading to a change of consciousness (Ren et al., 2020). Figure 1 summarizes the possible pathophysiological mechanisms leading to SAE.

## Synaptic dysfunction in SAE

**Figure 2:**
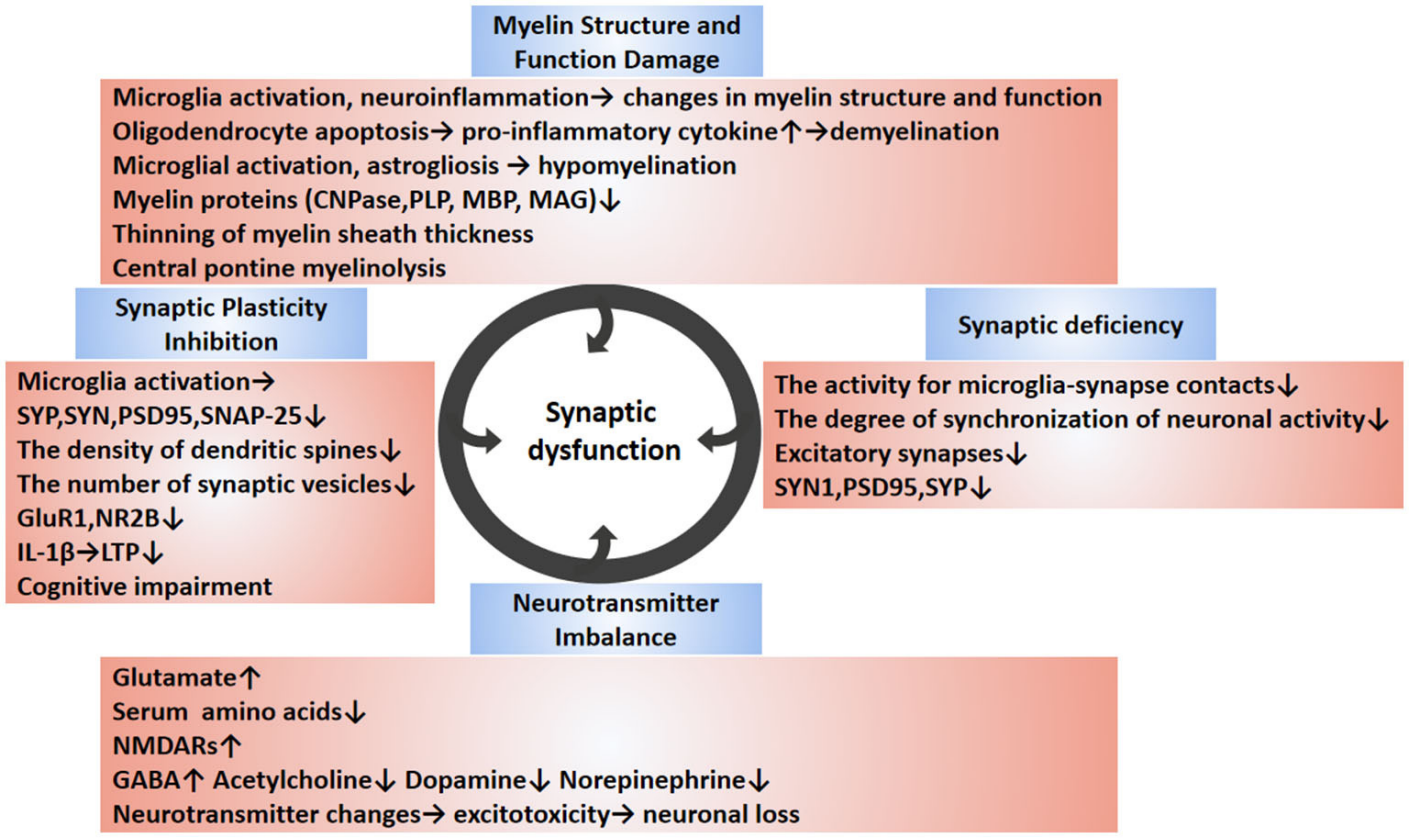
Synaptic dysfunction of SAE. The synaptic dysfunction of SAE is multifactorial mediated (circles indicate). SAE-induced neuroinflammation can cause cellular changes in brain tissue, such as the release of pro-inflammatory cytokines from activated glial cells, resulting in synaptic and neuronal damage. The abnormal synapse-related changes during SAE are diverse and include reduced synapse number, reduced expression of synapse-associated proteins, abnormal neurotransmitters, inhibition of synaptic plasticity, and changes in myelin. Disruption of synapses leads to inhibition of synaptic plasticity. Abnormalities in neurotransmitters can cause excitotoxicity and lead to neuronal death. Demyelination and hypomyelination can inhibit synaptogenesis, which can lead to neurological dysfunction. In conclusion, the causes of synaptic damage are multiple factors, and these synaptic abnormalities affect the synaptic transmission, ultimately leading to cognitive and memory impairment. The blue boxes describe relevant changes in synaptic dysfunction reported in SAE studies. The pink boxes outline the changes in specific molecules of action and their underlying pathological mechanisms. SAE, sepsis-associated encephalopathy; SYP, synaptophysin; PSD95, postsynaptic density protein 95; NR2B, N-methyl-D-aspartate receptor subunit 2B; GluR1, Amino-3-hydroxy-5-methy-4-isoxazole propionate (AMPA) receptor subunit; SNAP-25, synaptosomal associated protein of 25 kD; LTP, long-term potentiation; CNPase, 2′, 3′-cyclic nucleotide 3′-phosphodiesterase; MBP, myelin basic protein; PLP, proteolipid protein; MAG, myelin associated glycoprotein; C3a, complement 3 a; SYN1, synapsin 1; GABA, glutamate, g-Aminobutyric acid; NMDAR, N-methyl D-aspartate receptor.

Early cognitive decline in Alzheimer's disease has been reported to be associated with synaptic loss (Jamjoom et al., 2021). In a large proportion of patients with sepsis, cognitive dysfunction can reach the level of mild Alzheimer's disease; therefore, cognitive dysfunction due to sepsis may also be associated with synaptic loss (Annane and Sharshar, 2015). Although the relationship between synaptic loss and cognitive impairment in sepsis is unclear, there is evidence that what occurs in sepsis survivors is closely related to cognitive impairment with activated microglia and cytokine- mediated synaptic defects (Moraes et al., 2015). Therefore, the presence of long-term cognitive deficits in SAE patients may be closely related to synapse abnormalities. In the next section, we will focus on synaptic dysfunction, including synaptic plasticity inhibition, neurotransmitter imbalance, synaptic deficiency, and myelin damage caused by SAE (Figure 2).

### Synaptic plasticity inhibition

Synaptic plasticity refers to the characteristic that the number, structure, and strength of synaptic connections can be adjusted between neurons, which is the manifestation of learning and memory activities. Synaptic plasticity mainly includes long-term potentiation (LTP) and long-term depression (LTD), which represent enhancement and weakening phenomena in the synaptic signal strength between two neurons, respectively. Among them, LTP is generally regarded as one of the main molecular mechanisms constituting the basis of learning and memory. Thus, inhibition of synaptic plasticity can affect learning and memory function. Investigators observed that presynaptic proteins synapsin (SYN), synaptophysin (SYP), postsynaptic density protein 95 (PSD 95), and N-methyl-D-aspartate receptor subunit 2B (NR2B), Amino-3-hydroxy-5-methy-4-isoxazole propionate (AMPA) receptor subunit (GluR1) were downregulated, and hippocampal LTP inhibition in SAE animals (Zong et al., 2019b; Qin et al., 2021). Lin et al. found that microglia activation and inflammatory responses in neonatal rats injected with LPS reduced SYP and synaptosomal-associated protein (SNAP) expression, the density of dendritic spines, and the number of hippocampal synaptic vesicles (Lin et al., 2019). Therefore, it is speculated that LPS-induced microglia activation may lead to dysfunction of synaptic development and synaptic plasticity, and affect later intellectual development (Lin et al., 2019). Moreover, it was found that IL-1b caused LTP deficiency of the hippocampus associated with SAE-induced neuroinflammation, while the inhibition of IL-1β can alleviate cognitive deficiency (Imamura et al., 2011; Hoshino et al., 2021). Another pharmacological study also demonstrated that reducing neuroinflammation improved cerebral microvascular function, thereby preventing cognitive impairment during the acute phase of sepsis (Reis et al., 2017). Additionally, the use of selective M1 muscarinic acetylcholine receptor agonist or acetylcholinesterase blocker can also partially rescue LPS-induced LTP impairment, which demonstrates that enhanced cholinergic function restores deficits in synaptic plasticity (Zivkovic et al., 2015). These data support the idea that neuroinflammation is a critical neurological impairment mechanism due to SAE. This suggests that cognitive dysfunction in SAE may not be caused by a single factor, such as a cytokine or glial activation, but is the result of sepsis triggering a complex multifactorial interplay leading to a persistent neurological damage process. Normally, synaptic plasticity depends on the normal function of hippocampal synapses. Therefore, SAE-induced inhibition of synaptic plasticity may be due to neuroinflammation mediated-synaptic damage.

### Myelin structure and function damage

Myelin which is a membranous structure wrapped outside the axons of neurons can insulate and improve the conduction velocity of nerve impulses. Synapse and myelin sheath are key structures to ensure the normal function of neurons, and it is an important guarantee for the normal conduction of nerve impulses (Mamoon et al., 1977). The investigators found that dysmyelination can inhibit synapse formation leading to synaptic dysfunction and neurological deficits while promoting myelination can improve synaptic development and nerve conduction function in mice after hypoxia (Wang et al., 2018).

The neuroinflammatory response mediated by microglia activation can lead to changes in myelin structure and function in the corpus callosum region of mice (Aryanpour et al., 2017). Liu and colleagues used LPS to stimulate the activation of microglia and collected the conditioned medium to incubate oligodendrocytes *in vitro*, and found that oligodendrocytes apoptosis significantly increased (Liu et al., 2018). Inhibition of this oligodendrocyte apoptosis reduces pro-inflammatory cytokine levels and rescues the neuroinflammation-mediated demyelination (Liu et al., 2018). This result demonstrates that glial activation and neuroinflammation may be the cause of myelin sheath injury, thus leading to synaptic and neurological damage. Recent studies have shown that neonatal sepsis-induced periventricular white matter (PWM) damage, which is related to microglial activation, astrogliosis, and resultant hypomyelination (Xie et al., 2016; Han et al., 2017; Huang et al., 2020; Jiang S. et al., 2021). Huang et al. demonstrated that complement 3 a (C3a), which is associated with neuroinflammation, caused a decrease in the expression of myelin proteins including PLP (proteolipid protein), MBP (myelin basic protein), MAG (myelin associated glycoprotein) and CNPase (2′, 3′-cyclic nucleotide 3′-phosphodiesterase), and a thinning of myelin sheath thickness (Huang et al., 2020). What's more, central pontine myelinolysis (whose pathological features include marked demyelination of the central pontine with fewer axons and neurons) has been found in patients with SAE, although the relationship between central pontine myelinolysis and SAE is currently unclear (Wilson and Young, 2003). Overall, myelination is one of the necessary conditions for synaptic and neural function development, so myelin sheath structure and function damage may lead to synapse loss or synaptic dysfunction on inflammatory pathological backgrounds such as SAE.

### Neurotransmitter imbalance

Neurotransmitters are well-known as chemical substances that transmit signals between pre- and post-synaptic neurons. The imbalance of neurotransmitters also seems to be involved in the development of SAE. The accumulation of glutamate which is the most abundant excitatory neurotransmitter in the brain, may lead to neuronal excitotoxicity (Moraes et al., 2021). The septic animal model experiments confirmed the imbalance of glutamate, and inhibiting glutamate release can reduce cognitive impairment and improve the survival rate of septic animals (Freund et al., 1985; Toklu et al., 2009; Kurtz et al., 2019). A previous study found that serum levels of amino acids required for neurotransmitter synthesis were reduced in SAE patients (Basler et al., 2002). This may lead to abnormalities in the neurotransmitter system in patients with SAE. Changes in neurotransmitters (including GABA, acetylcholine, dopamine, and norepinephrine) have been reported in SAE patients or animals (Han et al., 2001; Basler et al., 2002; Tauber et al., 2021). Furthermore, glutamate regulates microglia. The investigators downregulated LPS-induced pro-inflammatory cytokine expression in microglia by inhibiting NMDA receptors (Wu et al., 2018). A pharmacological trial also demonstrated that neurological symptoms were improved and survival was enhanced in cecal ligation and puncture (CLP) treated rats through the use of glutamate release inhibitor (Toklu et al., 2009). Another work also found that in SAE mice, the glutamate excitotoxicity induced by glutamate receptor N-methyl-D-asperate receptor subunit 2 (NR2)excess was attenuated by inhibiting ferroptosis, thereby protecting synaptic and neuronal integrity (Xie et al., 2022). All of these findings imply that disorders of neurotransmitters and the expression of their receptors play a key role in SAE. Thus, excitotoxicity caused by changes in these neurotransmitters can lead to neuronal loss, which may account for the clinical and electrophysiological features of the SAE (Mazeraud et al., 2020).

### Synaptic deficiency

Cognitive function is closely related to the abundance of synapses (Manabe and Heneka, 2022). Studies have confirmed that synapse density is reduced in the hippocampus of patients with cognitive impairment such as early-onset Alzheimer's disease and mild cognitive impairment, so it is speculated that cognitive impairment in SAE patients may be caused by synapse loss (Terry et al., 1991; Scheff et al., 2007; Vanhaute et al., 2020). Both age-related spines loss and reduced axospinous synapse density can be observed in the monkey prefrontal cortex (Dumitriu et al., 2010). Similarly, age-related loss of hippocampal synapses was found in aging mice (Shi et al., 2015). During normal development, microglia can sculpt neural circuits by promoting the synaptic formation of neurons and selectively eliminating or phagocytosing synapses (Schafer et al., 2012). Recent studies have shown that although microglia are able to recognize and eliminate excitatory synapses, no increase in synaptic pruning by microglia was found in SAE (Manabe et al., 2021). However, it appears that microglia recognize and prune excitatory synapses labeled by complement factor 3 (C3) at some point in time, while inhibitory synapses do not change. Furthermore, Akiyoshi et al. find that the direct contact between resting microglia and synapses can lead to increaes in contacted synaptic activity and neuronal activity (Akiyoshi et al., 2018). When microglia are activated by LPS (or partially removed), the activity for microglia-synapse contacts is no longer increased, and the degree of synchronization of neuronal activity is also decreased. So, in the case of neuroinflammation, activation of microglia may lead to synaptic deficits. In the SAE animal model, a significant decrease in synaptic associated proteins including synapsin 1 (SYN1), postsynaptic density protein 95 (PSD95), SYP and NMDA receptors (NR2B), the dendritic spine density and neuron loss could be observed (Xu et al., 2019; Li C. et al., 2020). Often, the reduction of the synaptic proteins indirectly reflects synaptic dysfunction. These data show abnormal changes in synapses and neurons in SAE. In contrast, treatment with electroacupuncture (EA) protects against SAE-induced cognitive impairment by inhibiting synaptic damage and neuron loss (Li C. et al., 2020). Recently, it is found that the expression of synapse-associated protein (related to synapse formation and function) is downregulated in patients with delirium induced by infection, which means that synapses in SAE patients may be reduced (Peters Van Ton et al., 2020). Notably, the conflicting data have been found in some animal experiments, probably because of differences in the CLP surgical approach as well as the LPS injection protocols. For instance, there is no loss of neurons and dendritic spines, and synaptic changes were found (Chen et al., 2012, 2014; Zhang et al., 2017; Beyer et al., 2020). Regrettably, there are currently no clinical data on synapse density in SAE patients. Therefore, we speculate that the cause of SAE-induced synaptic dysfunction or synaptic deficiency could be a problem in which module during synapse formation (e.g., synapse pruning) or synapse maintenance? Is it possible that the SAE-induced neuronal impairment and synaptic dysfunction are caused by abnormal synapse or synaptic formation? Next, we will discuss synapse/synapse formation in normal and SAE conditions.

## Synapse and synapse formation in SAE

### Synapse and synapse formation

It is well known that synaptically released neurotransmitters (e.g., glutamate and GABA) regulate neuronal excitability and synaptic transmission and maintain the excitatory/inhibitory balance of neural circuits (Akerman and Cline, 2006; Dorrn et al., 2010; Sun et al., 2010; Yizhar et al., 2011). This abnormality of excitation/inhibition balance is considered to be the possible mechanism of a variety of neurodevelopmental diseases, such as epilepsy, autism spectrum disorders (ASD) and schizophrenia (Cline, 2005; Dudek, 2009; Lisman, 2012). Some research data suggest that IL-1b may alter the synaptic strength of central GABAergic synapses, leading to the cognitive dysfunction observed in SAE (Serantes et al., 2006). Increasing evidence suggests that LPS caused distinct changes in synaptic protein content, spine dynamics and delayed reduction of excitatory synapses (Weberpals et al., 2009; Kondo et al., 2011; Manabe et al., 2021). Together, these data all demonstrated the synaptic alterations during SAE. Whether this synaptic alteration (e.g., synaptic deficiency as described previously) is due to a decrease in synapse formation (or synaptogenesis) or an increase in synapse loss is not known. Synapse formation is a basic process that constitutes the brain's neural circuit, but its mechanism is poorly understood. Throughout the process of synapse formation, the synaptic connection achieves a relative balance between excitability and inhibition through synaptic specialization, maturation, and synaptic pruning in order to maintain normal physiological functions (West and Greenberg, 2011). In conclusion, synapse formation is not limited to neurodevelopment, it is a lifelong process. When lesions occur in the nervous system, neuronal death may occur, leading to synaptic dysfunction such as in Alzheimer's disease. Synapse formation is a rather complex process involving a large number of factors. Thus, we particularly focus on the key players associated with the synaptic formation in the following.

### Factors mediating synapse formation

#### Microglia and complement system activation

As mentioned earlier, in the early postnatal period, the number of synapses formed in the brain is much higher than that in adulthood, but approximately half of all synapses can remain and function during development while the rest are eliminated, which is called “synaptic pruning.” Only after undergoing the process of pruning and reconstruction can more accurate synaptic connections be formed in the brain (Bilimoria and Stevens, 2015).

Numerous studies have confirmed the important role of microglia in synapse formation and elimination, regulating synaptic plasticity, promoting the maturation of synapses and neural circuits, and maintaining the stability of the environment in the CNS (Miyamoto et al., 2016; Weinhard et al., 2018; Andoh and Koyama, 2021). The synaptic pruning function mediated by microglia can clear the synapses that are less stimulated by signals, that is, “weaker” synapses, while retaining the synapses that are often stimulated by signals, that is, “stronger” synapses during development. In pathological conditions, its synaptic pruning function can clear dead cells and myelin fragments, and also wrap around bare axons to form myelin sheath and promote synapse and myelin sheath regeneration during brain injury repair (Tremblay et al., 2010). Microglia activation and alterations of the spine triggered by LPS indicate that microglia involvement in synaptic remodeling and this pathological mechanism may also underlie cognitive dysfunctions after SAE (Kondo et al., 2011).

Hypothalamus contains a large number of neuroendocrine cells, which play an important role in regulating normal physiological function and homeostasis by secreting neurohormones. In CLP-treated animals, sepsis caused massive apoptosis of vasopressinergic neurons and impaired secretion of arginine vasopressin (AVP) in the hypothalamus were found to be closely associated with microglia activation and BBB leakage (Da Costa et al., 2017). Another study also showed that hypothalamic dysfunction due to AVP damage observed in septic surviving rats was associated with hypothalamic synaptic defects (Santos-Junior et al., 2018)). Hence, impaired AVP which plays the role of antidiuretic and vasoconstrictive effects secretion may be the cause of hypotension shock in sepsis patients.

The complement system is a critical component of innate immunity and serves many important functions in CNS, and is implicated in disease processes (Veerhuis et al., 2011; Ratajczak et al., 2018). The complement C3 which cleavage product C3a binds to the G protein-coupled receptor of C3a receptor (C3aR) is the central molecule of the complement system and plays a pivotal role in the immune system (Lian et al., 2015; Hong et al., 2016; Lohman et al., 2017). The increasing study has suggested that complement C3/C3a receptor signaling, a key component of innate immune pathogen defense, plays an important role in many neurological disorders (Garred et al., 2021). C3a receptor expressions in microglia were specifically up-regulated and triggered by LPS. C3a receptor antagonist attenuated LPS-induced hippocampal neuroinflammation and inhibited synapse-related protein loss, contributing to improved cognitive function (Li C. et al., 2020; Garred et al., 2021). Furthermore, the complement receptor 3 (CR3/CD11b-CD18/Mac-1) as a key molecular mechanism underlying the engulfment of developing synapses, is significantly expressed on microglia and its ligand and localized to synaptically-enriched regions. The disruption of CR3/C3 signaling was specific to microglia in the CNS and resulted in sustained defects in synaptic connectivity (Stevens et al., 2007; Schafer et al., 2012). Mice with fewer microglia (Il34–/– mice) or deficiency in complement C3 or C3a receptors were protected from viral-induced synaptic terminal loss (Vasek et al., 2016; Litvinchuk et al., 2018; Xiong et al., 2018). These evidence indicate that microglia activation and complement C3/C3a receptor signaling may be associated with spine remodeling after LPS treatment (Weberpals et al., 2009; Kondo et al., 2011; Manabe et al., 2021). Microglial CR3 activation can trigger LTD, which may lead to memory damage and synaptic disruption, under the synergistic effect of hypoxia and LPS (Zhang et al., 2014). These data demonstrate that the complement system-dependent microglia activation plays an important role in synaptic functional regulation and remodeling synapses in development and disease. However, to our knowledge, there is no evidence that activation of microglia and complement system is directly involved in SAE pathology. In the below sections, we discuss the synaptic proteins associated with the synaptic formation in SAE, summarized in Table 1.

**Table 1:** Summary of synaptic proteins.

**Synaptic** ** protein**	**Research subject (Method)**	**Outcome**	**References**
Neurexin-3a	Cultured rat embryonic neurons	Neurexin-3α↓ synapse development alteration	Gresa-Arribas et al., 2016
Nrxn1 SNAP-25 SYN-2 NLGN SHANK3	Primary human neuronal-glial (HNG) cell cocultures after exposure to Bacteroides fragilis lipopolysaccharide (BF-LPS)	Nrxn1↓ SNAP-25↓ SYN-2↓ NLGN↓ SHANK3↓	Zhao et al., 2019
RPTP γ	LPS treated mice	The astrocytic expression of RPTPγ↑	Lorenzetto et al., 2014
RPTPβ/ζ	LPS treated mice LPS stimulated BV2 microglial cell cultures	Inhibition of RPTPβ/ζ modulates LPS induced responses *in vivo* and in *vitro*	Fernandez-Calle et al., 2020
MANF	Marine invertebrate *Litopenaeus vannamei* 293T cell culture	Shrimp MANF is associated with RPTP to mediate negative regulation of ERK activation and Dorsal expression shrimp RPTP-S overexpression could switch shrimp and human MANF mediated ERK pathway activation to inhibition	Luo et al., 2022
SYP SNAP-25	LPS treated rats	SYP↓ SNAP25↓ NMDAR↓ the number of synapse and synaptic vesicles↓ appeared swollen dendritic spines of pyramidal neurons↓ proinflammatory mediators↑	Lin et al., 2019
SYP	CLP treated rats CLP treated mice	□SYP↓ cognitive deficits □hypothalamic SYP↓ Bcl2↓ cleaved caspase- 3↑ AVP secretion ↓ □SYP↓ TNF-α↑ cognitive impairment cAMP response element binding protein (CREB) ↓ insulin signaling disrupted	Schwalm et al., 2014; Neves et al., 2018; Santos-Junior et al., 2018
STEP	LPS treated mice	STEP↓ CREB/BDNF↓PSD95↓ STEP Inhibition reversed memory impairment	Zong et al., 2019a
SYP PSD95	CLP mice neuronal cultures with conditioned medium derived from cultured astrocytes (ACM) and microglia (MCM) pregnant Swiss Webster (SW) mice with Klebsiella pneumoniae injection neonates	□SYP↓ PSD95↓ synaptic deficit LPS-MCM : synapses↓ LPS-ACM : synapses↑ □SYP↓ PSD95↓ motor impairment learning, and memory impairments	Moraes et al., 2015; Granja et al., 2021
TSP TSP-1	Septicemia patients and septic patients with MODS TSP1-/- mice with CLP or *E. coli* injection	□TSP on platelets↑ platelets activated TSP-1/- survival ↑ after CLP or *E.coli* injection □FcγR-mediated phagocytosis of macrophages in TSP-1-/- mice ↑	Gawaz et al., 1997; Mcmaken et al., 2011

Nrxn1, neurexin1; SNAP-25, synaptosomal associated protein of 25 kD; SYN-2, synapsin-2; NLGN, neuroligin; SHANK3, the SH3-ankyrin repeat domains3; RPTP γ, Receptor protein tyrosine phosphataseγ; RPTPβ/ζ, Receptor protein tyrosine phosphatase β/ζ; MANF, mesencephalic astrocyte-derived neurotrophic factor; SYP, synaptophysin; STEP, striatal-enriched protein tyrosine phosphatase; PSD95, postsynaptic density protein 95; TSP, thrombospondin; TSP-1, thrombospondin-1; LPS, lipopolysaccharide; CLP, cecal ligation and puncture; RPTP, Receptor protein tyrosine phosphatase; NMDAR, N-methyl D-aspartate receptor; TNF-α, tumor necrosis factor- alpha; AVP, arginine vasopressin; CREB/BDNF, response element binding protein (CREB)/brain-derived neurotrophic factor (BDNF). ↓: increase; ↑: decrease.

### Synaptic adhesion molecules and proteins mediating synapse formation

#### Neurexins and ligands

Synapse formation is a dynamic process, which is characterized by the interactions between pre- and post-synaptic neurons. Synaptic adhesion molecules (SAMs) accumulate at pre- and postsynaptic sites, initiate synapse formation, and play an important role in regulating synaptic transmission and synaptic plasticity (Jang et al., 2017; Sudhof, 2018; Südhof, 2021; Yuzaki, 2018; Kim et al., 2021). The presynaptic SAMs are mostly present in excitatory and inhibitory synapses, like neurexins which are a family of SAMs involved in synapse formation and maturation, and leukocyte antigen–related (LAR)-type phosphotyrosine phosphatase receptors (PTPRs) (Sudhof, 2017; Cornejo et al., 2021; Fukai and Yoshida, 2021). In contrast, postsynaptic SAMs are more diverse as ligands for these presynaptic SAMs and are often specific for excitatory or inhibitory synapses. This evidence suggests that the transmembrane signals generated by SAMs mediate synapse formation and organization (Sudhof, 2018). However, it is not very clear which signal pathways regulate synapse formation. Neurexins (Nrxns) are predominantly expressed in neurons as a family of presynaptic transmembrane proteins (Ushkaryov et al., 1992). Further, it has been found that neurexin1 (Nrxn1) was also expressed in astrocytes by single-cell RNA sequencing (Gokce et al., 2016). Currently, neurexins ligands include neuroligins (NLGNs), Leucine-rich-repeat transmembrane neuronal proteins (LRRTMs), latrophilins, dystroglycan, Brain Angiogenesis Inhibitors (BAIs), kainate receptor subunit (GluK), neurexophilins, calsyntenins and cerebellin1-3 (cbln1-3) (Sudhof, 2017, 2018). These findings reveal that neurexins can play a role by combining different ligands to organize complex interaction networks and their signaling pathways.

NLGNs are classical ligands of neurexins. Different neuroligins have different localization at synapses. Early studies found that a knockout of single a- Neurexin decreased the survival rate of experimental mice, while knockout of all a - Neurexins can significantly attenuate the synaptic transmission (Missler et al., 2003). All b-conditional knockout of neurexins can also lead to attenuation of synaptic transmission in excitatory neurons, but has no obvious effect on the survival (Anderson et al., 2015). In cortical PV- positive interneurons, conditional knockout of all Neurexins coding genes leads to loss of synapses and weakening of the synaptic strength (Chen et al., 2017). Another study found that neurexin-3a is a novel autoantigen of antibodies in patients with autoimmune encephalitis. In embryonic neurons of rats exposed to patients' IgG, a specific decrease in neurexin-3a levels, as well as a reduction in the total number of synapses, was observed. This result suggests that neurexin-3a may affect the synapse formation (Gresa-Arribas et al., 2016). In addition, it was found that the expression of five synaptic proteins includingNrxn1, synaptosomal associated protein of 25 kD (SNAP-25), the phosphoprotein synapsin-2 (SYN-2), NLGN, and the SH3-ankyrin repeat domains3 (SHANK3) was significantly decreased in human neuronal-glial (HNG) cell co-cultures after Bacteroides fragilis lipopolysaccharide (BF-LPS) treatment (Zhao et al., 2019). This result indicates that abnormal changes of these pre- and post-synaptic proteins may lead to synaptic dysfunction in the pathological process of SAE, but it does not indicate that synaptic adhesion molecules or their ligands play a crucial role in the synaptic formation.

### Receptor protein tyrosine phosphatases

Receptor protein tyrosine phosphatases (RPTPs) are key regulators of the neuronal morphogenesis (Andersen et al., 2001). It has been shown that two isoforms of RPTPg expressions were increased on astrocytes after LPS treatment, and RPTPg was also found to be strongly positive in activated astrocytes in Alzheimer's disease (Lorenzetto et al., 2014). RPTPb/z is a target receptor for the cytokines pleiotrophin (PTN) and midkine (MK) and regulates peripheral and central immune responses (Herradon et al., 2019). In addition, the RPTPb/z plays a role in the potentiation of microglial activation and in the microglia neuron communication during LPS-induced neuroinflammation (Fernandez-Calle et al., 2020). In a recent marine invertebrate study, RPTP was confirmed to be a key component of extracellular shrimp mesencephalic astrocyte-derived neurotrophic factor (MANF) mediated inhibition of the ERK pathway and participation in inflammatory regulation (Luo et al., 2022). These findings all emphasize the role of RPTPs during the neuroinflammatory process, although the exact mechanism is unclear.

In summary, these SAMs can affect synapse production, but it seems that none of them is necessary. These data suggest that synapse formation may be mediated by multiple SAMs at the same time. Multiple SAMs form a complex interaction network, which can activate different signaling pathways or common important signaling pathways (Sudhof, 2017). In addition, different SAMs are responsible for executing different aspects of synapse formation, and each independently plays a role in regulating the establishment and maintenance of synapses (Sudhof, 2018). Due to the lack of experimental evidence on adhesion molecules of synaptic cells during SAE, it is necessary to conduct more systematic research in combination with other research methods.

### Other synapse-associated proteins

#### SYP and PSD95

Synaptic associated proteins mainly include SYP in the presynaptic membrane, and postsynaptic marker postsynaptic plasticity protein PSD95). These proteins play different roles in the regulation of synaptic structure and function. SYP, a specific marker of synaptic plasticity, is involved in synaptic vesicle transport and neurotransmitter release, and can specifically reflect the number, distribution and density of synapses (Griva et al., 2017). Some studies have shown that the abnormal expression level of SYP can be observed in neuroinflammation and hypoxia-ischemia (Rao et al., 2012; Griva et al., 2017). The SYP and SNAP-25 protein expression was markedly decreased in optical density, and the number of synapses and synaptic vesicles was reduced and appeared swollen after LPS administration (Lin et al., 2019). Reduced SYP and cognitive deficits found in in sepsis-survivor animals can be prevented by treatments that reduce acute brain inflammation and oxidative stress (Schwalm et al., 2014). In CLP-treated animals, reduced expression of SYP associated with synaptic plasticity and concomitant cognitive deficits can be observed in the hippocampus (Neves et al., 2018). PSD95 as a major regulator of synaptic maturation, is involved in the glutamatergic transmission, synaptic plasticity, and dendritic spine morphogenesis during neurodevelopment. It has been shown that PSD95 dysfunction may alter synaptic plasticity at the dendritic spines that contribute to the malformations of the synapse associated with neurological disorders (Coley and Gao, 2018; Kinjo et al., 2018). A recent report showed that inhibition of the striatal-enriched protein tyrosine phosphatase (STEP) signaling pathway significantly improved SAE-induced memory impairment by increasing the expression of PSD95 (Zong et al., 2019a). Other studies have also demonstrated that both SYP and PSD95 levels were significantly decreased in the hippocampus of the SAE mice model (Moraes et al., 2015; Santos-Junior et al., 2018). Another report about gestational sepsis suggests that maternal sepsis can induce an inflammatory response accompanied by a reduction of SYP and PSD95 levels and leads to cognitive and behavioral alterations in offspring (Granja et al., 2021). It is well known that synaptic damage will cause the impairment of cognition and memory. Indeed, there is evidence that reductions in synaptic markers may contribute to cognitive decline in neurodegenerative and neuropsychiatric disorders (Rao et al., 2012; Hong et al., 2016). Therefore, cognitive and memory dysfunction in SAE may be caused by synaptic damage (e.g., synaptic alterations of PSD95 and SYP).

### Thrombospondin-1

The thrombospondins (TSPs) are a family of oligomeric multidomain glycoproteins that are distributed throughout vertebrates and interact with the extracellular matrix, growth factors, cell surface and proteases (Mosher and Adams, 2012). Accumulating data show that TSPs promote new synapse formation and regulate synaptic functions, mainly mediated by the interaction between TSPs and related neuronal receptors in the developing CNS (Risher and Eroglu, 2012; Ferrer-Ferrer and Dityatev, 2018). Currently, it is generally believed that TSPs are through receptor α2δ-1 and NLGN to regulate synaptogenesis and participate in synaptic remodeling through extracellular matrix proteins and cell surface receptors after injury (Wang et al., 2012; Risher et al., 2018). After CNS injury, TSP1 which is a member of the thrombospondin family can interact with the increasing transforming growth factor (TGF)-b1 to inhibit the release of IL-10 while possibly promoting synapse formation at the neuromuscular junction (Packard et al., 2003; Feng and Ko, 2008). Moreover, it is found that the expression of TSP on the platelet surface is increased in sepsis patients, and polymorphisms of TSP are involved in the development of sepsis-related organ failure (Gawaz et al., 1997). Research also proves that the increased TSP expression can be observed in critically ill patients with severe sepsis, and TSP-1 deficiency was protective from the mortality of sepsis and increased bacterial clearance in two models of septic mice (Gawaz et al., 1997; Mcmaken et al., 2011).

### Signaling pathways for synapse formation mediated by SAMs

Synapse formation is a precisely regulated process. Studies suggest that transmembrane signals involved in SAMs can regulate synapse formation. However, it is still unclear which signal pathways mediated by SAMs regulate synapse formation (Sudhof, 2018). A recent study reported that synapse formation is driven *via* multiple signaling pathways such as PKA-, JNK-, and AKT-mediated intracellular signaling pathways by SAMs (Jiang X. et al., 2021). Although the interaction with transsynaptic adhesion molecules and the downstream processes are largely unknown, these signaling pathways provide important clues for us to understand synapse formation (Sudhof, 2018; Jiang X. et al., 2021). The c-Jun N-terminal kinases (JNKs) that members of the mitogen-activated protein kinase (MAPK) family, regulate important physiological processes, including embryonic development and neuronal functions (Zeke et al., 2016). The JNK/MAPK signaling pathway plays a role in synapse formation during development and nervous system function in *C. elegans* (Andrusiak and Jin, 2016). Recent studies have shown that the JNK pathway is an important component of the heterologous synapse formation process capable of assembling pre and postsynaptic specialization mechanisms (Jiang X. et al., 2021). Crawley et al. found the MIG-15/JNK-1 MAPK pathway can restrict both glutamatergic synapse formation and learning in *C. elegans*, suggesting that the JNK/MAPK signal pathway may also play a similar role in the formation of synapses in vertebrates (Crawley et al., 2017). Although recent studies have also revealed that inhibiting JNK signaling pathway can protect the mice against LPS-induced septic mice (Mo et al., 2021; Nie et al., 2021). However, there are no relevant reports or data on the role of the JNK/MAPK signaling pathway in the formation of synapses under SAE or sepsis pathological conditions.

## Conclusions

SAE is a common severe complication of sepsis, mainly manifested as acute brain dysfunction. To date, there is no special treatment to prevent or reverse SAE. At present, the mechanisms involved in the pathophysiology of SAE, such as neuroinflammation, neurovascular injury, glial activation, and so on, have been reported. The result of these pathological changes and a series of cascade reactions in SAE are synaptic dysfunction, dysregulated neurotransmission and neuron loss, which ultimately leads to neurological damage. Although synapse changes dynamically during development, abnormal changes (too much or too little) in the number of synapses are closely related to many neuropsychiatric diseases. Furthermore, the structural and functional damage of synapses and the abnormalities in the whole process of synapse formation (including synaptic pruning) and synaptic maintenance can lead to synaptic deficits and neurological dysfunction. Therefore, we try to find evidence and relevant clues of abnormal synapse/synapse formation in SAE and explore its molecular mechanism. Taken together, studying the synaptopathy in SAE may bring us a new perspective and direction to understand and explore the pathological mechanism of SAE.

## Author contributions

CT and YJ collected the literature and wrote the manuscript. HW conceived the topic, wrote and edited the manuscript. All authors contributed to the article and approved the submitted version.

## Funding

This work was supported by the National Natural Science Foundation of China (81673417).

## Conflict of interest

The authors declare that the research was conducted in the absence of any commercial or financial relationships that could be construed as a potential conflict of interest.

## Publisher's note

All claims expressed in this article are solely those of the authors and do not necessarily represent those of their affiliated organizations, or those of the publisher, the editors and the reviewers. Any product that may be evaluated in this article, or claim that may be made by its manufacturer, is not guaranteed or endorsed by the publisher.
